# Histological and Immunohistochemical Characterization of the Tibial ACL Remnant: Implications for Ligament Healing

**DOI:** 10.3390/medicina62020407

**Published:** 2026-02-20

**Authors:** Sorin Florescu, Blidişel Iulian Alexandru, George Andrei Drăghici, Dragoş Vasile Nica, Boru Casiana, Cosmin Grațian Damian

**Affiliations:** 1 Discipline of Orthopedics-Traumatology, Department XV, “Victor Babeș” University of Medicine and Pharmacy Timișoara, Eftimie Murgu Square No. 2, 300041 Timișoara, Romania; 2 Department of Surgery I–Clinic of Surgical Semiotics & Thoracic Surgery, Center for Hepato-Biliary and Pancreatic Surgery, “Victor Babeș” University of Medicine and Pharmacy Timișoara, Eftimie Murgu Square No. 2, 300041 Timișoara, Romania; 3 Research Center for Pharmaco-Toxicological Evaluations, Faculty of Pharmacy, “Victor Babeș” University of Medicine and Pharmacy Timișoara, Eftimie Murgu Square No. 2, 300041 Timișoara, Romania; 4 Faculty of Pharmacy, “Victor Babeș” University of Medicine and Pharmacy Timișoara, Eftimie Murgu Square No. 2, 300041 Timișoara, Romania; 5 The National Institute of Research—Development for Machines and Installations Designed for Agriculture and Food Industry (INMA), Bulevardul Ion Ionescu de la Brad 6, 077190 București, Romania; 6 Dental Research Center Using Conventional and Alternative Technologies, “Victor Babeș” University of Medicine and Pharmacy Timișoara, Eftimie Murgu Square No. 2, 300041 Timișoara, Romania; 7 Faculty of Medicine, “Vasile Goldiș” Western University of Arad, Bulevardul Revoluției 94, 310025 Arad, Romania

**Keywords:** anterior cruciate ligament, reconstruction, tibial stump, CD34, S100, neurofilament protein, vimentin, actin

## Abstract

*Background and Objectives*: The pathways mediating the beneficial effect of tibial stump preservation for anterior cruciate ligament (ACL) reconstruction remain insufficiently clarified. This study investigated key vascular, neural, and stromal aspects of cellular remodeling processes occurring across lesion stages in tibial remnant pre-reconstruction. *Materials and Methods*: Biopsies were obtained from 25 patients undergoing arthroscopic ACL reconstruction (paired free-end and tibial insertion sampling) and 10 from quasi-normal, macroscopically intact ligaments (controls). We evaluated intergroup differences in microvascular density using a *t*-test. Group comparisons for angiogenesis (CD34), neural components (S100, neurofilament-associated proteins—NFAPs), and stromal activation (vimentin and actin) were conducted using Chi-square or Fisher’s exact tests. *Results*: ACL remnants revealed a significantly higher microvascular density (37 ± 2.3 vs. 18 ± 3.2 vessels/mm^2^, *p* < 0.001), in addition to a markedly increased prevalence of synovial angiogenesis (90% vs. 20%, *p* < 0.001), stellate stromal cells (94% vs. 10%, *p* < 0.001), and CD34-positive fibrocytes (92% vs. 10%, *p* < 0.001) compared to control tissues. Elevated intraligamentous neovascularization (with borderline significance) was also found in these tissues (38% vs. 0%, *p* = 0.045). Both injured and control ACLs showed widespread S100-positive neural fibers, suggesting maintained Schwann cell integrity despite ligament disruption. In contrast, control ligaments showed a substantially richer NFAP+ neural network, particularly in small-caliber fibers and free nerve endings, pointing to preferential vulnerability of small-caliber neural elements during ACL rupture. Vimentin expression changes—from homogeneous fibrocytic staining to diffuse reticular overexpression in fibrotic lesions—were accompanied by the emergence of stellate myofibroblast-like cells, supporting advanced stromal remodeling. Absent in controls, actin immunoreactivity increased with lesion severity, indicating a progressive myofibroblastic response driven by perivascular cells during ligament remodeling. *Conclusions*: The tibial ACL remnant is a biologically active, compartmentalized repair niche driven by coordinated vascular, neural, and stromal responses, with reparative activity concentrated at the synovial–epiligament interface. These findings support the biological rationale for preserving tibial remnant for ACL reconstruction.

## 1. Introduction

Anterior cruciate ligament (ACL) reconstruction is one of the most common surgical orthopedic interventions [1,2]. Aiming to restore the native ligament biomechanics and reduce the risk of graft failure, this procedure typically uses bone–patellar tendon–bone, hamstring tendon, and quadriceps tendon as autograft sources [2,3,4]. The newly formed ligamentous structure integrates into the articular environment, transforming from tendon to ligament and serving as a functional biomechanical restrictor [5]. Despite this, biological integration of the graft is deficient compared to the healing of natural ligaments. One approach to improve postoperative evolution is to retain the residual tibial stump [6,7]. In this context, it is important to note that ACL remnants not only have a good regenerative potential, but also contain viable nerve endings [8,9]. These data indicate that the tibial remnant has the ability to regenerate vessels, fibroblasts, and new tissue while possessing intact or partially intact sensory nerve fibers. The maintenance of the mechanically nonfunctional tissue may hence contribute to motor function through better proprioception after ACL reconstruction.

The healing of the ACL tibial stump and autograft ligamentization involves complex biological processes orchestrated by a large number of proteins. It is known that torn ACL stumps are rich in progenitor cells and fibrocytes with high content of CD 34—a transmembrane phosphoglycoprotein central to neovascularization, fibrocyte recruitment, and tissue regeneration [10,11,12]. However, its precise role in graft integration remains to be established. The members of the S-100 family of calcium-binding proteins (produced, among others, by Schwann cells and glial cells) can act as signaling molecules for trauma-induced inflammation (e.g., the S100A8/A9 proteins), as well as markers for nerve function and proprioception (e.g., the S100B protein) [13]. Although evidence supports that the tibial stump can retain mechanoreceptors and nerve fibers positive for S-100 [13], its involvement in ligament healing is not well defined. The most abundant components of neuronal cytoskeleton, neurofilament-associated proteins (NFAP) serve as pertinent markers of neuroaxonal integrity or damage, and hence of remaining nerve fibers. Clinical data hint at incomplete nerve regrowth into ligament grafts [14,15], but it remains unclear whether and how a robust reinnervation process can be achieved after ACL reconstruction. A cytoskeletal marker of stromal activation, vimentin exerts multiple physiological roles in healthy ligaments, including mechanical resilience, as well as cell adhesion and migration [16,17]. This intermediate filament protein is often considered just a cell marker, with its functional impact on signaling and tissue regeneration during ligament healing remaining unclear. Actin is another indicator of stromal/fibrotic activation, an evolutionarily conserved protein linked to all phases of wound healing [18,19]. Despite its obvious importance, specific insights on actin role in ACL graft healing derive from general wound-healing biology rather than ligament-specific data. These proteins capture different aspects of healing pathways and reveal inter-pathway dependencies.

Recent histopathological investigations have highlighted variable neo-vascularization and cellular changes in ACL remnants across different age groups and injury durations. For example, Sahu et al. reported differential histological features in ruptured ACL remnants in a prospective observational study, suggesting that specific microstructural conditions may be associated with tissue remodeling processes [20]. A recent descriptive analysis reported persistence of immature and intermediary blood vessels in ACL remnants, suggesting angiogenic activity even months after injury [21]. However, most available studies have investigated vascularity, innervation, or stromal activity in isolation, although there is growing interest in remnant-preserving ACL reconstruction. There is also a lack of integrative histological analyses that simultaneously address angiogenesis, fibroblastic activation, and neural integrity within distinct anatomical compartments of the tibial ACL remnant. Moreover, spatial differences between the free end and the tibial insertion of the remnant remain insufficiently characterized.

In this context, the aim of the present study was to conduct a combined histological and immunohistochemical assessment of vascular, stromal, and neural markers in paired tibial anterior cruciate ligament (ACL) remnant samples, in order to delineate compartment-specific remodeling patterns associated with ligament rupture. Specifically, we investigated the involvement of these proteins in the remodeling processes occurring within the tibial stump following ACL reconstruction. Our hypothesis was that the tibial remnant of ruptured ACLs represents an active neuro-stromal niche rather than an inert debris, with regenerative features concentrated at the synovial–epiligament interface. Analysis of histoarchitectural organization around the remaining stump post-rupture provides relevant information on the interrupted processes, as well as the local repair potential. On the other hand, histology and immunohistochemistry on tibial-stump biopsies (free end and insertion/base) resolve meso- to micro-scale events, namely angiogenesis (CD34), stromal activation/fibroplasia (vimentin), myofibroblastic differentiation (actin), and reinnervation (S100 and NFAP). Paired site sampling from the free end and the tibial insertion of the ligament captures spatial gradients of revascularization and innervation, while quasi-normal ACL samples (controls) anchor specificity. Together, this sequence allows us to connect vascular, neural, and fibroblastic remodeling in the tibial stump—a necessary chain of evidence for understanding integration and failure risks after ACL reconstruction.

## 2. Materials and Methods

### 2.1. Study Design

This study was an observational, cross-sectional, single-site study conducting histological and immunohistochemical analysis of human ACL tissue samples. The study population included patients admitted to the Arad County Emergency Hospital, Clinic of Orthopaedic and Traumatology, for knee arthroscopic procedures between February and October 2025. All participants signed informed consents for participation and the study protocol was approved by the Institutional Ethics Committee at this hospital (approval number 92/20 January 2025).

### 2.2. Participants and Eligibility Criteria

Biopsy (ligament) specimens were obtained from 25 patients with ACL rupture. For each patient, two samples were collected from the tibial stump—one from the free end and one from the insertion site (base). Control samples were obtained from ten cases with macroscopically intact (quasi-normal) ACLs without rupture or inflammation, removed during arthroscopic debridement for technical reasons. The primary inclusion and exclusion criteria for injured ACL and control ACL groups are given in Table 1.

The demographic and clinical characteristics of the study population are provided in Table 2. Given the exploratory nature of the study, we did not conduct formal sample size determination or a priori power calculation. In fact, the study sample was defined by the availability of eligible human ACL remnant tissue obtained during arthroscopic surgery.

### 2.3. Histological and Immunohistochemical Methods

Biopsies were washed with normal saline (0.9% NaCl) and fixed in 10% buffered formalin (pH 7.2). Small tissue blocks (10 × 10 × 3 mm) were maintained for 24 h in 15–25 milliliters (mL) of 10% buffered formalin. These samples were processed by dehydration in alcohol solutions of 70% (30 min), 80% (30 min), 95% (30 min), and 99% (60 min × 2); clarification in xylene, 2–3 changes of 20–30 min each; and embedding in paraffin at ~55–60 °C (4 × 60 min) using a Shandon Excelsior AS Tissue Processor (Thermo Fisher Scientific Inc., Waltham, MA, USA). Serial sectioning (15 sections/block, 5 microns thick) was performed with a semiautomatic microtome Shandon Finesse ME (Thermo Fisher Scientific Inc., Waltham, MA, USA). The sections were mounted on capillary-gap slides (DakoCytomation A/S, Glostrup, Denmark) and placed in a thermostat at 60 °C for 60 minutes (min) and then at 37 °C for 24 h. Deparaffinization of histological sections was carried out in a thermostat at 58 °C for 60 min. The slides were placed in xylene baths (2 × 5 min), and then in ethanol baths in decreasing concentrations 100%, 95%, 80%, and 75% (5 min each). The samples were rehydrated with deionized water (2 × 10 min). Tissue sections were stained with hematoxylin–eosin (H&E) and Masson’s trichrome for general histological evaluation and collagen visualization, respectively. Gordon & Sweets method (standard silver impregnation method) was used to identify reticular fibers [22,23].

Immunohistochemical analysis was conducted on 3 mm thick, paraffin-embedded ACL sections [24]. The sections were deparaffinized in xylene at a thermostat (58 °C, 60 min), followed by xylene baths (2 × 10 min) and sequential baths in ethanol 99.5%, 96%, 80%, and 70% (10 min each) at room temperature. Rehydration followed the procedure described above. Endogenous peroxidase activity was blocked by incubation with hydrogen peroxide 3% (5 min) and deionized water (5 min). After washing in phosphate-buffered saline (PBS), nonspecific binding was minimized by incubation with normal goat serum (Vector Laboratories, Burlingame, CA, USA) for 20 min at room temperature. Sections were incubated with the appropriate primary antibody, followed by a secondary detection system based on horseradish peroxidase (HRP) and 3,3′-diaminobenzidine (DAB; Agilent Technologies, Santa Clara, CA, USA) as the chromogen. CD34 was detected with the clone QBEnd/10 (ready-to-use; Biogenex, San Ramon, CA, USA) and the immunoreactivity was visualized with the EnVision™ Detection System (Agilent Technologies, Santa Clara, CA, USA), with hematoxylin counterstaining. For each sample, microvascular density was assessed by manually counting immunoreactive microvessels in three representative high-power fields within areas of highest vascular density and expressed as vessels per square millimeters (mm^2^).

The S100 protein was identified by immunohistochemistry using a FLEX polyclonal rabbit anti-S100 antibody (ready-to-use, code GA50461-2; Agilent Technologies, Santa Clara, CA, USA), as previously described in the literature [25,26], with the antibody being diluted 1:100. Vimentin was determined with the clone V9 (ab8069, diluted 1:100; Abcam, Cambridge, UK). Neurofilament (NF200, clone N52.1.7, mouse monoclonal, ready-to-use, code PA0371; Leica Biosystems, Newcastle Upon Tyne, UK) was used as the antibody for NFAP. For these proteins, immunostaining was carried out using the LSAB2 + HRP System kit (Agilent-Dako, Santa Clara, CA, USA). Antigen retrieval was performed prior to immunostaining by heating the sections in citrate buffer (10 mM, pH 6.0) using a microwave oven for 20 min (CD34 and S100) or 30 min (vimentin and NFAP) at 95–99 °C, followed by cooling at room temperature. The examination was performed using the Eclipse600 Nikon optical microscope (Nikon Corp., Tokyo, Japan), whereas the images were taken using the Coolpix950 digital camera Nikon Corp., Tokyo, Japan. The microscopic image analysis was performed using the LuciaNet program (Nikon Corp., Tokyo, Japan).

### 2.4. Statistical Analysis

Differences in demographic and clinical characteristics of the study participants were conducted with independent *t*-tests in the case of continuous variables (age, height, weight, body mass index, time from injury to surgery). Categorical variables (sex, smoking status) were analyzed using the Chi-square (χ^2^) test, or Fisher’s exact test when expected cell frequencies were below 5. Comparison between groups in terms of microvascular density was conducted using again an independent *t*-test. Exploratory correlations between microvascular density and age, height, weight, and body mass index were assessed using Spearman’s rank correlation coefficients. Differences in frequency distribution of synovial angiogenesis, intraligamentous blood vessels, stellate stromal cells, and CD34^+^ fibrocytes in control (quasi-normal) versus injured tendons were analyzed as categorical variables for demographic and clinical characteristics. A *p* value less than 0.05 was considered significant. All data were processed with the Statistica 10 software (StatSoft Inc., Tulsa, OK, USA).

## 3. Results

The injured and control ACL groups were comparable with respect to demographic and clinical characteristics. We observed no significant differences in terms of age (*t*(14.3) = −0.82, *p* = 0.423), body height (*t*(14.8) = −0.42, *p* = 0.683), body weight (*t*(15.1) = 1.36, *p* = 0.185), and body mass index *(t*(14.9) = −0.63, *p* = 0.532). Sex distribution (*t*(14.8) = −0.42, *p* = 0.683) and smoking status (χ^2^(1) = 0.01, *p* = 0.912) were also similar between groups. These data showed that injured and control ACL groups were well matched across key demographic and clinical parameters, indicating a homogeneous study population and minimizing the potential influence of baseline confounders on ACL-related outcomes.

### 3.1. CD34 Expression

**Figure 1:**
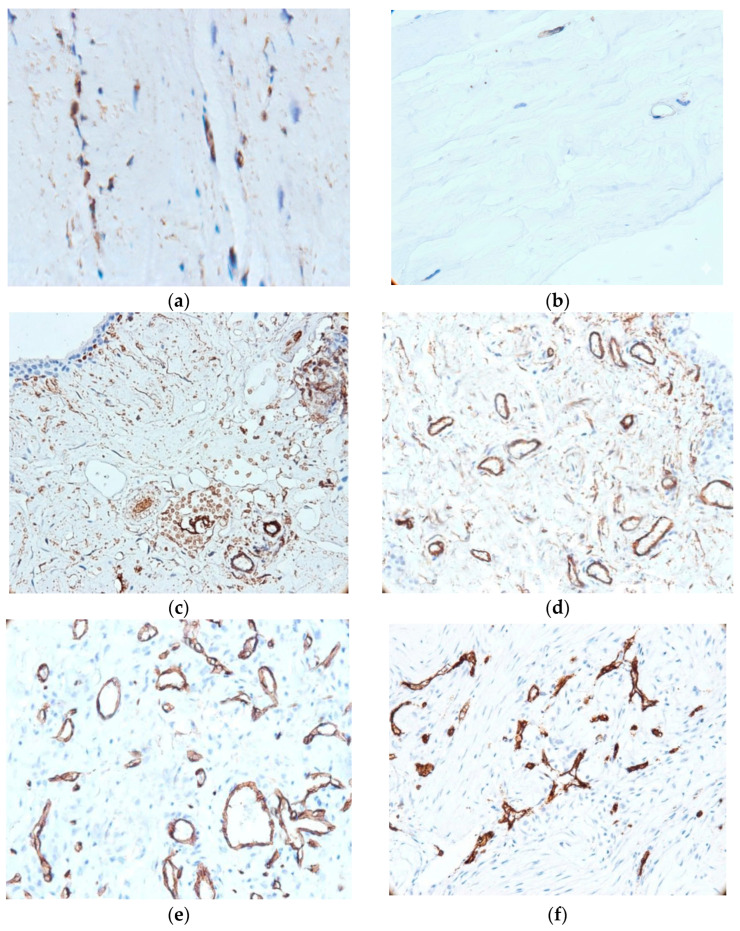
Histological and immunohistochemical features of ligament and synovial tissue. (**a**) CD34^+^ fibrocytes in morphologically altered ligament tissue (400×); (**b**) CD34: weak/negative immunoreaction in control tissue (400×); (**c**) small blood vessels concentrated in the vicinity of the covering epithelium (200×); (**d**) vascular network of the synovium, mostly with permeable lumen (400×); (**e**) developed microvascular network, with evolving angiogenesis; (**f**) immature blood vessel network (200×).

Figure 1 illustrates the histological and immunohistochemical differences between control and injured ligament/tendon tissues, highlighting CD34^+^ fibrocyte distribution and associated angiogenic changes. In ligament samples, fibrocytes were predominantly observed in the peripheral region adjacent to the synovium, whereas the central areas contained fewer cells. Control specimens revealed fibrocytes that were either CD34-negative or weakly immunoreactive (Figure 1a). In contrast, CD34^+^ fibrocytes were predominantly localized within areas of structural alteration in the ligament (Figure 1b). In addition, samples from the injured tendons consistently showed CD34^+^ fibrocytes interspersed among collagen fibers (Figure 1c–e) with cells arranged in bi- or quadrigeminate groups—similar to tendon structure.

Microvascular density showed variability between samples, being significantly higher in tendon lesions (*t*-test, 37 ± 2.3 vs. 18 ± 3.2 vessels/mm^2^, *t*(11.6) = 17.87, *p* < 0.001). Exploratory Spearman correlation analyses did not reveal significant associations between microvascular density and patient age (ρ = −0.16, *p* = 0.421), body mass index (ρ = 0.10, *p* = 0.618), body height (ρ = −0.08, *p* = 0.712), or body weight (ρ = 0.13, *p* = 0.553). Synovial neovascularization was primarily observed adjacent to the covering epithelium and was commonly associated with CD34^+^ fibroblasts. Blood vessels showed different size, shape, and wall thickness, consistent with an active neovascularization process in cases with ACL lesions. We frequently observed marked hyperplasia of stellate and spindle stromal CD34^+^ cells—in 20 out of 50 cases (40%)—with the synovial surface showing layering and disruption of its structural integrity. Approximately one-quarter of cases (11 out of 50 biopsies) showed intraligamentous neoangiogenesis in the areas of injury. These capillary-type vessels, small in caliber and with a narrow lumen, were sometimes detected inside the injured ligament (26%, 13/50 cases). These vascular structures tended to form irregular networks that separate the bundles of collagen fibers within the ligament (Figure 1f). We note the coexistence of two types of vessels (immature and intermediate) in 17 cases (34%); this signals an evolving angiogenesis process.

The data on frequency of synovial angiogenesis, intraligamentous blood vessels, stellate stromal cells, and CD34^+^ fibrocytes in control versus injured tendons are provided in Table 3. Synovial angiogenesis was observed at a significantly higher frequency in injured tendons than in control tendons (χ^2^(1) = 20.11, *p* < 0.001). The number of stellate stromal cells and CD34^+^ fibrocytes was significantly greater in injured tendons (χ^2^(1) = 31.69 and 28.36, respectively; *p* < 0.001 for both). Blood vessels within the ligament were observed only in injured tendons, with this difference also showing borderline significance (χ^2^(1) = 3.47, *p* = 0.047).

### 3.2. Neural and Stromal Immunohistochemical Findings

**Figure 2:**
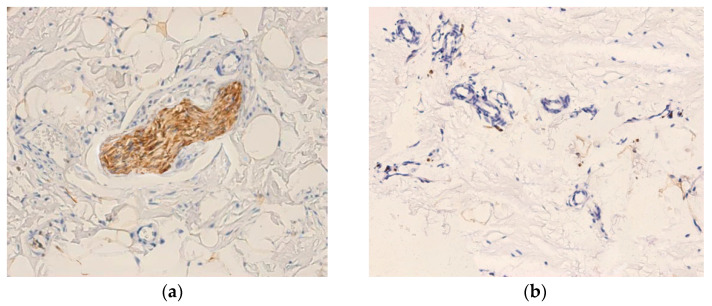

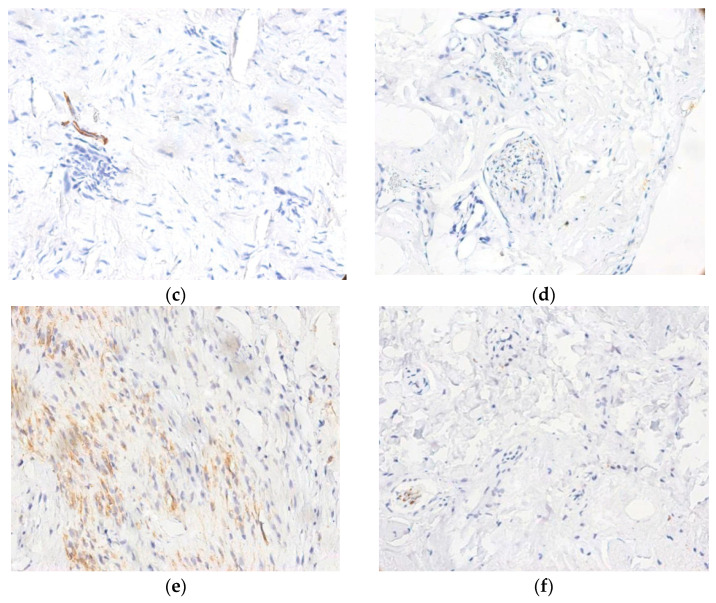
Expression of S100 (**a**–**c**) and NFAP (**d**–**f**) proteins in control ligament, disrupted ligament tissue, and synovial tissue. (**a**) Intense cytoplasmic reaction in S100+ Schwann cells associated with large-caliber nerve fibers (400×); (**b**) Presence of S100+ positive nerve endings located in close proximity to small blood vessels (400×); (**c**) Control ligament with numerous large-caliber S100+ nerve fibers showing intense positivity (400×); (**d**) NFAP+ large-caliber nerve fibers with focal medium-to-low cytoplasmic staining in Schwann cells (400×); (**e**) NFAP+ expression in control ligament, with abundant small-caliber nerve fibers and free nerve endings (400×); (**f**) NFAP+ immunoreactivity in the affected ligament, demonstrating a markedly reduced density of small-caliber nerve structures vs control ligament areas (400×).

Immunohistochemical expression patterns of S100 and NFAP proteins are illustrated in Figure 2. The S100 and NFAP expressions were detected in both synovial tissue and injured ACLs. We detected the expression of S100 in 37 out of 50 cases (74%), and NFAP expression in 31 out of 50 cases (62%). Although S100 was present in a higher proportion of cases compared to NFAP, this difference did not reach statistical significance (Chi-square test, χ^2^(1) = 1.66, *p* = 0.203). The former protein was abundantly expressed in small nerve fibers of the synovial tissue, with staining intensity ranging from strong to moderate-low. Large fibers showed a homogeneous expression restricted to Schwann cell cytoplasm (Figure 2a), while small fibers displayed a heterogeneous distribution with moderate-low S100 intensity. A similar expression was observed in free nerve endings, some of which were located adjacent to small blood vessels (Figure 2b).

Interestingly, S100+ ligament tissue occurred in both small and large nerve structures, but at low frequency (≈2–3 small fibers and 1–2 large fibers per microscopic field at 400× magnification). The expression of S100 showed a heterogeneous pattern, being strong in large fibers and moderate-to-high in small fibers. The staining was typically homogeneous in Schwann cell cytoplasm. Overall, S100 intensity and distribution appeared comparable in ruptured and control (quasi-normal) ligaments, with a slight tendency toward higher levels in the latter (Figure 2c).

The expression of NFAP was markedly lower compared to S100 (Figure 2d). Ligamentous regions showed an increase in the number of NFAP+ free nerve endings in areas, corresponding to 3–4 positive endings per microscopic field at 400×, or areas containing both free nerve endings and small-caliber nerve fibers arranged in clusters (Figure 2e). In large-caliber nerve fibers, the NFAP expression was not only weaker (vs. S100) but also predominantly confined to Schwann cell cytoplasm, with medium-to-high staining intensity (Figure 2f). Control ligament areas contained a higher number of NFAP+ nerve fibers (free nerve endings and small-caliber fibers) compared with areas of disrupted ligament tissue and synovium.

**Figure 3:**
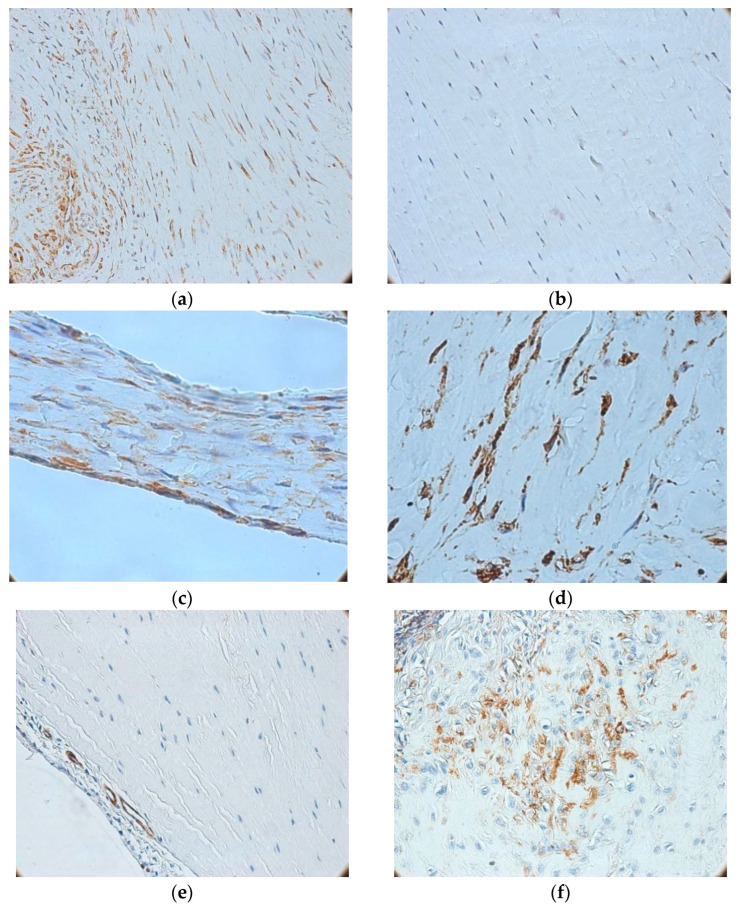
Immunohistochemical profiles of vimentin (**a**–**d**) and actin (**e**,**f**) in ACL tissues (**a**) Positive vimentin immunoreactivity in control ligaments (100×); (**b**) Negative vimentin reaction in ligament with minor microscopic lesions (100×); (**c**) Severe ligament lesion showing compact fibrosis and intense reticular vimentin staining (400×); (**d**) Spindle-shaped cells with cytoplasmic extensions, high density, and strong reaction (400×); (**e**) Negative actin reaction in control ligaments (100×); (**f**) Actin-positive perivascular cells in synovial vessels (400×).

The expression patterns of vimentin and actin, as assessed via immunohistochemistry, are shown in Figure 3. Vimentin expression was positive in all biopsies, with internal controls represented by adipocytes, vascular smooth muscle cells, mesothelial cells, endothelial cells, fibroblasts, and fibrocytes. Evaluation of vimentin expression primarily identified three major patterns: (*i*) intense, homogeneous positivity in fibrocytes from control (quasi-normal) ligaments (Figure 3a); (*ii*) reduced and heterogeneous expression in interfibrillar fibrocytes from lesions with mild-to-moderate collagen disorganization, with peripheral positivity (Figure 3b); and (*iii*) overexpression in perivascular and perineural cells of the adjacent synovium. In severe lesions—identified in 14 out of the 50 ligament biopsies with lesions (28%)—a distinct pattern emerged, characterized by extensive collagen disorganization and replacement with compact fibrotic tissue. In these cases, vimentin staining demonstrated strong diffuse positivity, with a reticular distribution (Figure 3c). Moreover, inflammatory infiltrates exhibited increased density but reduced vimentin intensity—in contrast with samples obtained from mild-to-moderate ligament lesions. In both moderate and severe lesions, many vimentin-positive intraligamentary cells displayed a stellate, branched morphology resembling myofibroblasts (Figure 3d), whereas fusiform fibrocytes were rare or absent, prompting additional evaluation with smooth muscle actin immunostaining.

Actin imunoreactivity was detected in all samples, being most prominent in the synovium. However, intraligamentary positivity was observed in only about one-quarter of ACL biopsy specimens with lesions (15/50 cases, 30%). Control ligaments were entirely negative (Figure 3e), whereas small- and medium-caliber synovial vessels demonstrated perivascular positivity with low microvascular density (Figure 3f). In cases with preserved fibrillar structure, focal clusters of actin-positive cells were detected. These cells were typically spindle-shaped, exhibiting cytoplasmic fibrillar immunostaining, and were mainly located in the synovium—similar to vimentin-positive cells. Most moderate lesions (24/26 cases, ≈92%) revealed small fascicles of intraligamentary actin-positive cells with granular cytoplasmic staining, frequently associated with chronic inflammatory infiltrates. Extensive fibrotic lesions showed strong, multifocal or diffuse actin expression overlapping vimentin-positive regions, highlighting myofibroblastic transdifferentiation of perivascular cells, particularly pericytes.

## 4. Discussion

The results of our study provide pertinent immunohistological evidence that the ACL remnant is as a biologically active, compartmentalized repair organ with a synovial-driven stromal, vascular, and neural response that preserves sensory potential and offers tangible targets for regenerative and neurotrophic interventions. We demonstrate that reparative activity (CD34^+^ fibrocytes, angiogenesis, Schwann cell survival) is concentrated at the synovial–epiligament interface, not uniformly across the ligament—suggesting that this interface represents a preferential site of reparative activity after ACL injury. Literature data are compatible with the presence of a small number of fibrocytes with very weak or no CD34 immunoreactivity in healthy ligament [27], but a marked increase in CD34-positive fibrocytes in injured specimens, primarily in damaged intraligamentous regions [16,28,29]. Our results are consistent with these findings, with significant rise in CD34^+^ stromal cells and their presence among the collagen fibers of injured tendons providing support for an association between tendon injury and the presence of fibrocytes/stromal progenitors in injured regions—these cells migrate to injured tissue and differentiate into collagen-producing cells [30,31]. Indeed, the CD34-expressing subset increased type II collagen production, angiogenesis, and osteogenesis in an ACL reconstruction model in mice [32]. Furthermore, these cells were observed to concentrate in peripheral region near the synovium, while the ligament mid-substance remained relatively hypocellular. This peripheral accumulation supports that the synovial–epiligament interface as a key region associated with reparative cell presence post-injury [12,33]. We also note that the arrangement of these cells in pairs or groups of two or four along collagen bundles resembles native tendon cell rows [34], potentially reflecting expansion or alignment of new fibroblasts attempting to reconstruct the ligament microstructure.

Neovascularization was another prominent feature separating injured from control ACL. Damaged ligaments showed a significantly higher proportion of samples exhibiting synovial angiogenesis and a significant, greater than twofold rise in microvascular density. These new vessels were often seen in regions adjacent to the synovial lining and were accompanied by clusters of CD34-positive fibroblasts/fibrocytes with a thickened, multilayered synovial lining instead of the normal single thin layer. These findings are consistent with an enhanced angiogenic response accompanied by cellular hyperplasia—processes routinely associated with the proliferative phase of wound healing following ligament injury [35]. There is also evidence for an active angiogenic process. Thus, we observed a heterogeneous morphology (size, shape, wall thickness) of blood vessels in injured cases, with immature thin-walled vessels coexisting with more “intermediate” caliber vessels. These new capillary networks extended beyond the synovium in injured ligaments whereas control ligaments showed no intra-ligamentous blood vessels. The presence of persistent vascular structures and stromal cells in ACL remnants aligns with broader literature describing angiogenic activity and extracellular matrix remodeling in injured ligament tissue [36]. Taken together, these changes suggest that ligament injury is associated with a pronounced angiogenic shift, particularly at the synovial insertion and, to a lesser extent, into the ligament core, presumably to support the increased metabolic demands of healing tissue. These results position CD34^+^ cells as structurally involved in ligament remodeling rather than merely passive vascular-associated cells.

In response to peripheral nerve injury, Schwann cells undergo dedifferentiation, proliferation, and migration. This transition towards a regenerative phenotype is accompanied by persistent S100 expression [37], with activated S100-positive cells being pivotal to neuronal survival and regeneration [38,39,40]. In fact, repair Schwann cells are known to form Büngner bands and maintain these guidance structures for prolonged periods in denervated nerve stumps, thereby ensuring a living conduit for potential axonal regrowth [41,42,43]. The very high prevalence of S100 expression observed here in injured ligaments indicates persistence of Schwann cell–associated structures after rupture. This suggests relative preservation of the glial scaffold in the traumatized joint environment [44,45,46]. On the other hand, the substantial, heterogeneous S100 expression in small nerve fibers of the synovial tissue adjacent to the ACL remnant suggests that this tissue is enriched in Schwann cell–associated elements within a neurovascular niche [47]. This finding is also consistent with a variable but active glial/nerve fiber response—likely reflecting differential nerve fiber activation and remodeling in the post-injury joint microenvironment. The pattern seen in large intraligamentous nerves, with a homogeneous S100 expression confined to the Schwann cell cytoplasm, is consistent with either healthy myelinating Schwann cells or activated, post-injury Schwann cell forming a guidance sheath.

NFAP serves as an indicator of the severity of acute axonal damage [48]. In this context, an important morphological finding of our analysis is the expression pattern of NFAP within large-caliber nerves, with markedly lower immunoreactivity compared to S100 and signal limited to the Schwann cell cytoplasm. Since NFAP primarily stains the axon cylinder under normal physiological conditions, the latter event may reflect a major cellular event. Indeed, it is well established that Schwann cells shift their phenotype and have been reported to produce NFAP messenger RNA upon acute axonal disconnection. This morphological alteration is characteristic to the Schwann cell dedifferentiation, a process associated with the subsequent nerve repair response [49,50,51,52]. The observed pattern—high S100 staining and NFAP confinement within the glial sheath—provides evidence that the large-caliber axons previously ensheathed by Schwann cells (likely large proprioceptive mechanoreceptors) underwent complete Wallerian degeneration. It is hence plausible that the Schwann cell structure survived the trauma and transitioned toward a reparative phenotype, but the primary axonal content was lost.

The small-caliber neural structures, by contrast, revealed a more robust pattern of NFAP preservation. We identified a greater number of NFAP-positive free nerve endings in quasi-normal ligament areas, which is congruent with literature data [45]. This quantifiable persistence of free nerve endings (which generally represent small, unmyelinated nociceptive and fine sensory fibers) is compatible with a differential sensitivity to trauma based on nerve caliber. Thus, small-caliber sensory fibers may be more resilient or better nourished and sustained by the residual surrounding tissue compared to the larger, specialized encapsulated mechanoreceptor axons, which display signs of definitive degeneration (NFAP confinement). This pattern of differential preservation may be relevant for functional recovery. The high concentration of NFAP+ free nerve endings in the control (quasi-normal) tissue provides a putative anatomical substrate for retained somatosensory function, specifically basic position sense and protective nociceptive reflexes [52,53]. Since the survival of free nerve endings is crucial for knee joint position sense and the initiation of protective reflex arcs, their preservation is a key biological objective that justifies the retention of the remnant tissue. Collectively, these results are consistent with ACL remnants possessing the biological machinery required for potential regeneration, suggesting their possible relevance for neurotrophic intervention strategies.

ACL fibrocytes normally express vimentin [54]. The pattern seen in early lesions in interfibrillar cells—with lowered, heterogeneous, and peripherally confined expression of vimentin—is not explicitly described in orthopedical literature, but it is plausible: injury or collagen disruption may partially degrade fibrocytes or alter their phenotype, producing patchy vimentin staining. Related studies show that fibroblast activation in injury involves cytoskeletal remodeling (e.g., TGF-β1–driven fibroblast-to-myofibroblast conversion in tendon gratfs), which likely perturbs vimentin organization [55]. Thus, the observed decrease in vimentin expression may imply early fibrocyte stress or partial transition towards a myofibroblast state (which still expresses vimentin but in altered distribution). The strong, diffuse vimentin positivity with a net-like pattern detected in severe, extensive fibrotic lesions may reflect the presence of numerous vimentin-positive myofibroblasts or extracellular diffusion of vimentin epitopes from degenerating cells. Myofibroblasts co-express vimentin and α-smooth muscle actin [52], and therefore, it is likely that a dense fibrotic tissue (with many myofibroblasts) will induce pronounced immunopositivity for vimentin. Similar diffuse vimentin expression was reported in scar and fibrotic tissues (e.g., cardiac and lung fibrosis models), in which activated fibroblasts/pericytes predominates [54,55]. This “reticular” appearance, on the other hand, could be a novel observation; it may occur if dying fibrocytes release cytoskeletal proteins, coating the tissue. Interestingly, vimentin intensity decreased in inflammatory infiltrates of severe lesions. Although not well documented, this inverse relationship (more leukocytes but weaker fibroblast signal) may be compatible with fibroblast loss or dedifferentiation in the context of severe inflammation.

Beyond the ligament proper, synovial lining fibroblasts and vascular pericytes are known to constitutively express vimentin, with mesenchymal cells in joint tissues being vimentin-positive. Vimentin-expressing synovial fibroblasts were also reported in fibrotic tissues from arthritic joints [56]. In this context, the marked vimentin overexpression observed in perivascular and perineural regions of the synovium adjacent to the ACL is consistent with prior reports and supports the notion of localized fibroblastic activation. These findings may reflect the recruitment and/or activation of perivascular fibroblasts, pericytes, or bone marrow–derived fibrocytes in ACL pathology, indicating a potential association between ligament injury and coordinated responses within the surrounding synovial microenvironment [57,58].

Literature data hint at the presence of a baseline population of contractile fibroblasts in non-damaged ACL tissue. Thus, Murray and Spector described actin-positive cells (fibroblasts) in normal ACL at sites of fiber crimp [58], whereas Hasegawa et al. reported that almost one half of cells in young normal ACL are α-smooth muscle actin-positive [59]. In contrast, this protein was not detectable in our control samples. Differences in sampling or antibody sensitivity may help explain this discrepancy. Nonetheless, by standard understanding, ligament fibroblasts have minimal smooth-muscle actin unless activated [60]. The absence of these cells in controls may therefore imply truly quiescent fibroblasts, suggesting that any actin positivity in lesions is likely lesion-induced, not artifact.

Consistent with literature data [61], control (quasi-normal) synovial vessels showed α-smooth muscle actin in perivascular/muscular cells. The exclusive presence of focal clusters of spindle-shaped, cytoplasm-rich actin-positive cells in ligaments with preserved fibrillar structure, and especially in the synovium, may imply early myofibroblasts or pericytes responding to injury. Actin positivity became prominent in moderate lesions, with these cells showing a granular cytoplasm and being associated with chronic inflammation. This is consistent with the concept that injured tissue recruits fibroblasts that differentiate into α-smooth muscle actin-positive myofibroblasts under transforming growth factor beta 1 (TGF-β1) and inflammatory signals. Literature on tendon/ligament healing shows exactly this: for example, Xu et al. showed that TGF-β1 drives transition from fibroblast to myofibroblast and disordered collagen in ACL graft healing [54]. In addition, Hasegawa et al. found degenerated human ACLs to be enriched in α-smooth muscle actin-positive cells compared to aged normals [60]. Notably, we identified a strong and often diffuse actin staining in severe lesions, overlapping with vimentin-rich zones—a characteristic of mature myofibroblasts populating the scar [55]. One can hence reasonably infer that many fibroblasts/pericytes in these lesions have fully adopted a contractile phenotype. The localization (perivascular and perineural synovium) suggests involvement of pericytes: indeed, recent evidence identifies pericytes as a source of α-SMA+ myofibroblasts in fibrosis [57]. Taken together, these data are consistent with known fibrotic changes; that is, moderate and severe ACL lesions may resemble chronic scars with active myofibroblast populations.

Several limitations of the present study warrant consideration. First, its cross-sectional design precludes causal inference or determination of temporal remodeling dynamics. Second, the control group consisted of quasi-normal ACL tissue obtained during arthroscopic procedures, which may not fully capture the structural and cellular features of truly healthy ligaments. Although these samples lacked macroscopic and histological evidence of degeneration or inflammation, subtle alterations related to patient age, joint environment, or surgical indication cannot be entirely excluded. This limitation should be considered when interpreting comparisons between injured and control ACL tissues. Third, this study incorporates only histological and immunohistochemical observations, and therefore, no direct conclusions regarding clinical outcomes, biomechanical properties, or functional proprioception can be drawn from the present data. Finally, we used a relatively small sample size, and as an exploratory study, the current findings should be interpreted as descriptive and hypothesis-generating. Despite all these constraints, this study provides detailed compartment-specific histological evidence of coordinated vascular, stromal, and neural remodeling in the tibial ACL remnant.

Overall, our findings contribute to the literature across several dimensions. First, we demonstrate that reparative activity (CD34^+^ fibrocytes, angiogenesis, Schwann cell survival) is concentrated at the synovial–epiligament interface, not uniformly across the ligament—supporting this interface as a primary regenerative reservoir after ACL injury. Second, this study connects CD34^+^ stromal cell accumulation with active, heterogeneous neovascularization, showing a synchronized angiogenic–fibroblastic response rather than isolated vascular ingrowth. Third, our findings provide strong morphological evidence that large-caliber mechanoreceptive axons undergo Wallerian degeneration, while small-caliber sensory/free nerve endings are preferentially preserved, refining our understanding of post-injury sensory potential. Fourth, high, persistent S100 expression despite NFAP loss identifies surviving Schwann cells as a stable regenerative scaffold, not just passive remnants—an underemphasized concept in ACL pathology. Finally, we reveal stage-dependent, spatially heterogeneous cytoskeletal remodeling characterized by novel vimentin expression patterns and injury-induced emergence of focal actin-positive myofibroblast/pericyte-like phenotypes, indicating dynamic fibrocyte–myofibroblast transitions not previously well characterized in ligament tissue.

## 5. Conclusions

Our findings show that ACL injury is associated with a significant increase in microvascular density, synovial neovascularization, and prevalence of CD34^+^ fibrocytes and stellate stromal cells. This spatial coupling of angiogenesis and stromal activation is consistent with the concept of a synchronized vascular–fibroblastic response. The widespread presence of S100-positive Schwann cells in injured ligaments suggests relative preservation of glial elements following ACL rupture. In contrast, reduced NFAP expression was observed in injured tissues, particularly within large-caliber nerve fibers, consistent with axonal loss. These observations describe a neural microenvironment within the tibial remnant characterized by differential preservation of glial and axonal components. Ligament remodeling was also accompanied by a shift toward diffuse vimentin overexpression, lesion-dependent increase in actin immunoreactivity, and the emergence of myofibroblast-like cells. This integrative histological and immunohistochemical evidence supports the view that the tibial ACL remnant is not merely a passive byproduct of ligament rupture but a biologically active, compartmentalized repair niche. Vascular, neural, and stromal remodeling processes appear to occur in a coordinated and spatially organized manner, with reparative activity consistently preferentially concentrated at the synovial–epiligament interface. Preservation of the tibial ACL remnant during reconstruction may therefore have a sound biological rationale, with potential implications for enhancing graft integration, maintaining proprioceptive capacity, and improving long-term functional outcomes.

## Figures and Tables

**Table 1:** Inclusion and exclusion criteria.

Group	Inclusion Criteria	Exclusion Criteria
Injured ACL	aged 18 years or older clinically and imaging-confirmed anterior cruciate ligament rupture patients undergoing primary arthroscopic ACL reconstruction	prior history of knee surgery on the affected joint inflammatory joint disease advanced knee osteoarthritis systemic connective tissue disorders metabolic bone disease active joint infection
Control ACL	age 18 years or older macroscopically intact and functionally preserved ACL at arthroscopic inspection. undergoing knee arthroscopy for indications unrelated to ligament pathology	presence of any macroscopic, histological, or immunohistochemical evidence of ligament degeneration, partial rupture, inflammation, or synovial pathology prior history of knee surgery on the affected joint inflammatory joint disease advanced knee osteoarthritis systemic connective tissue disorders metabolic bone disease active joint infection

**Table 2:** Demographic and clinical characteristics of the study population.

Characteristic	Injured ACL	Control ACL
Age (years)	45.6 ± 7.8	48.1 ± 8.2
Sex (male/female), *n* (%)	15 (60%)/10 (40%)	6 (60%)/4 (40%)
Body height (cm)	173.1 ± 7.5	174.4 ± 8.0
Body weight (kg)	78.9 ± 11.2	73.3 ± 10.6
Body mass index (kg/m^2^),	25.3 ± 3.1	26.0 ± 2.9
Time from injury to surgery (months)	5 ± 2.3	—
Smoking status (yes/no)	7 (28%)/18 (72%)	3 (30%)/7 (70%)

Continuous variables (e.g., age, body weight) are given as mean with one standard deviation and. Categorical variables (e.g., sex, smoking status) are expressed as number of cases with the corresponding proportion (%) in parentheses. Relevant comorbidities included controlled arterial hypertension and mild metabolic abnormalities; no inflammatory joint disease, diabetes mellitus, or systemic connective tissue disorders were present in either group.

**Table 3:** Frequency of synovial angiogenesis, intraligamentous blood vessels, stellate stromal cells, and CD34^+^ fibrocytes in control versus injured ligaments.

	Synovial Angiogenesis		Blood Vessels		Stellate Stromal Cells		CD34^+^ Fibrocytes	
	Yes	No	Yes	No	Yes	No	Yes	No
Control tendon	2 (20%)	8 (80%)	0 (0%)	10 (100%)	1 (10%)	9 (90%)	1 (10%)	9 (90%)
Injured tendon	45 (90%)	5 (10%)	19 (38%)	31 (62%)	47 (94%)	3 (6%)	46 (92%)	4 (8%)

The data are given as absolute counts with corresponding percentages in parentheses. For each histological element, the table shows the number of cases (*n*) and the proportion (%) of positive (“Yes”) and negative (“No”) findings in both groups: control tendons (*n* = 10) and injured tendons (*n* = 50).

## Data Availability

All the data generated or analyzed in this study are included in this published article.

## Abbreviations

The following abbreviations are used in this manuscript:

ACL	Anterior cruciate ligament
CD34	Cluster of differentiation 34
DAB	3,3′-Diaminobenzidine
H&E	Hematoxylin and eosin
HRP	Horseradish peroxidase
IHC	Immunohistochemistry
NFAP	Neurofilament-associated proteins
TGF-β1	Transforming growth factor beta 1
PBS	Phosphate-buffered saline
S100	S100 calcium-binding protein family
α-SMA	Alpha-smooth muscle actin
